# The New Functional Hybrid Chaperone Protein ADGroEL–SacSm

**DOI:** 10.3390/molecules28176196

**Published:** 2023-08-23

**Authors:** Alisa Mikhaylina, Natalia Lekontseva, Victor Marchenkov, Viktoria Kolesnikova, Albina Khairetdinova, Oleg Nikonov, Vitalii Balobanov

**Affiliations:** Institute of Protein Research, Russian Academy of Sciences, Institutskaya Str. 4, 142290 Pushchino, Russia

**Keywords:** protein engineering, domain rearrangement, chaperone, hybrid protein

## Abstract

The creation of new proteins by combining natural domains is a commonly used technique in protein engineering. In this work, we have tested the possibilities and limitations of using circular homo-oligomeric Sm-like proteins as a basis for attaching other domains. Attachment to such a stable base should bring target domains together and keep them in the correct mutual orientation. We chose a circular homoheptameric Sm-like protein from *Sulfolobus acidocaldarius* as a stable backbone and the apical domain of the GroEL chaperone protein as the domain of study. This domain by itself, separated from the rest of the GroEL molecule, does not form an oligomeric ring. In our design, the hyperstable SacSm held the seven ADGroELs together and forced them to oligomerize. The designed hybrid protein was obtained and studied with various physical and chemical methods. Stepwise assembly and self-organization of this protein have been shown. First, the SacSm base was assembled, and then ADGroEL was folded on it. Functional testing showed that the obtained fusion protein was able to bind the same non-native proteins as the full-length GroEL chaperone. It also reduced the aggregation of a number of proteins when they were heated, which confirms its chaperone activity. Thus, the engineering path we chose made it possible to create an efficient thermostable chaperone. The result obtained shows the productivity of the way we chose for the creation and stabilization of oligomeric proteins.

## 1. Introduction

Combining and rearranging existing domains is the most prevalent way to obtain new proteins, both in nature and in protein engineering [1,2,3,4]. Many approaches to the design of such hybrid proteins have been developed and mastered [5,6,7]. Ring oligomeric proteins have a number of features that reduce their popularity for this method of construction. However, these features do not reduce the prospects for the engineering of such proteins. We became interested in the prospects and limitations of the use of hyperstable ring oligomer proteins as a scaffold for the design of new proteins. This was facilitated by two facts. On one hand, our institute has a good collection of ring-shaped oligomer proteins [8,9]. On the other hand, our laboratory actively researching the functions of GroEL [10] and other chaperones that are also ringed oligomers. At this junction, the work presented today was born.

Interest in the engineering and research of chaperones has been bolstered by the fact that such proteins are actively used in molecular biology and biotechnology [11]. Proteins with chaperone activity are often used to increase the efficiency of the production of target proteins in cells. For this, the co-expression of chaperone genes introduced on an additional plasmid or of producer strains with increased levels of their own expressions of chaperones is used [12]. Chaperones are often used to create fusion proteins. In such a hybrid, one component is the target protein, which is poorly produced in free form, and the second component is the chaperone itself. The most widely used variant of such a carrier chaperone is thioredoxin. The apical domain of the chaperone GroEL is also used. An interesting variant was proposed in Fedorov’s article, where a target toxic peptide was introduced into a break in the apical domain of GroEL (ADGroEL) sequence [13]. Also, chaperones are used in the refolding of proteins that cannot be obtained and purified in water-soluble form. The addition of chaperones as a component of the cell-free translation system makes it possible to increase the yields of active target proteins [14]. GroEL is probably the most studied chaperone in the world. However, there are a number of unanswered questions about its functioning. In particular, how important is its internal cavity? There are works that have shown the assistance of protein folding outside the cavity [10], but there are also works that have directly shown the presence of the peptide inside the cavity. In addition, as early as 1996, the preservation of the chaperone function in a separate monomeric ADGroEL has been shown [15]. The question arose: why, then, should it oligomerize? We decided to create an oligomer of ADGroEL. To carry this out, we took a homoheptameric Sm-like protein from *Sulfolobus acidocaldarius* (SacSm) as a basis and attached ADGroEL to each of its subunits. By creating such a hybrid protein (ADGroEL–SacSm), we ensured the oligomerization of ADGroEL and at the same time deprived it of its internal cavity as well as its binding sites for ATP and magnesium. According to current concepts, a significant part of the GroEL mechanism is responsible for the release of folded proteins [10,16]. In the future, the approach we have proposed will make it possible to isolate non-native proteins’ binding functions by separating them from other functions of GroEL.

Sm-like proteins present in bacteria, archaea, and eukaryotic organisms are RNA-binding proteins. They perform regulatory functions in the processes of transcription and translation; act as RNA chaperones that facilitate the interaction of small regulatory RNAs with mRNA; and take part in the processes of snRNA/rRNA/tRNA/mRNA splicing, histone maturation, and mRNA degradation [17]. However, the functions of these proteins were of little interest to us in this work. Of much greater interest was the possibility of choosing a protein with the required number of subunits in the ring and high structural stability [18]. The Hfq hexamer from *Pseudomonas aeruginosa* is able to withstand heating up to 100 °C and even higher [19]. Many of these proteins are also highly stable, and the more such domains are in our collection, the more diverse constructs we can obtain. This combination of properties makes Sm-like proteins a convenient module for protein engineering.

Analysis of the structure of SacSm shows that the target domain can be attached with a flexible linker at the N or C end, by lengthening the helix [20,21], or by one of the beta turns [22]. In this case, it is possible to provide both a flexible connection and a fairly rigid connection with a fixed position of the attached domain. To start our research, we chose the simplest option—a short 2xGly linker.

When designing a hybrid protein, we laid down an interesting principle that would allow us to further study its folding. The stability of the connected parts varied greatly. The SacSm structure had more stability than ADGroEL. That allowed us to fold/unfold only the ADGroEL by varying the conditions while maintaining the oligomeric structure of the entire protein or breaking it into monomers. The study of such a design will clarify one more question. The folding of the non-homologous domains of some multidomain proteins occurs independently [4,23,24,25]. On the other hand, for some proteins, it has been shown that excessively high similarities of amino acid sequences in domains connected in a single chain led to intraprotein aggregation and prevent protein folding in vitro [26,27]. Such problems are not observed in folding on the ribosome, since each domain folds, one after another, as biosynthesis proceeds. In our design, seven ADGroELs attached to a SacSm base were closely spaced and, in theory, could obstruct each other from folding. Was this true? The answer to this question was given by an experiment that studied the self-organization of a hybrid protein after its complete or partial unfolding.

Thus, in this paper, answers will be given to the following questions: (1) How suitable is SacSm as a scaffold for engineering? (2) What features of ADGroEL are retained while it retains its oligomeric form? (3) Will the mutual influence of domains on folding be critical? This article presents the first experiments in these areas, each of which undoubtedly deserves a separate deep study that will be carried out in the near future.

## 2. Results and Discussion

### 2.1. Stability and Folding of SacSm and ADGroEL Proteins

At the first stage, we assessed the stability and ability to oligomerize individual SacSm and ADGroEL proteins. We determined the presence of a secondary structure and its change under the action of denaturants with the CD method. The sizes of the formed particles in the native state and upon heating were determined with the DLS method. 

It has been shown that SacSm does not unfold in the presence of 6M urea and does not aggregate when heated to 80 °C in the absence of urea. At the same time, the particle size under native conditions corresponds to the size of a folded heptamer. 

**Figure 1:**
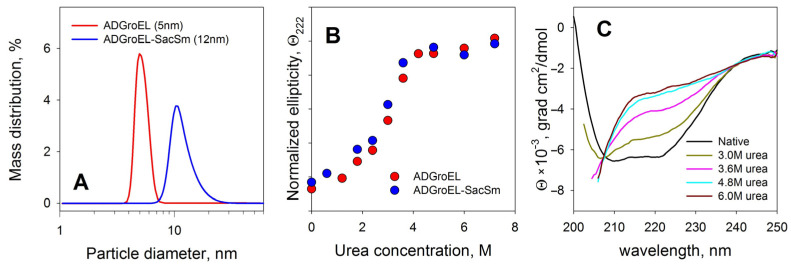
Determinations of the size of the resulting proteins with the DLS method (**A**), the stability of their structures upon unfolding with urea in the CD method (**B**), and the CD spectra of native fusion protein and spectrum changes during denaturation (**C**).

**Figure 2:**
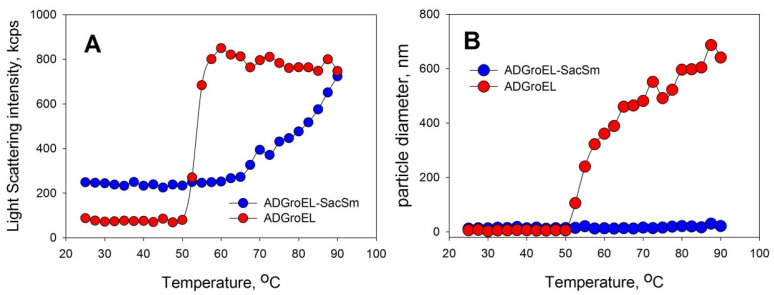
Determination of the aggregation abilities of ADGroEL and ADGroEL–SacSm with the DLS method. The protein concentration was 0.2 mg/mL. Heating was carried out at a rate of 1 °C per minute. The intensity of light scattering (**A**) and the average particle size in the solution (**B**) were determined.

The ADGroEL did not form oligomeric structures under the studied conditions. This is clearly indicated by the size corresponding to the folded monomer domain (Figure 1A). As the urea concentration increased, the ADGroEL underwent a cooperative denaturation transition, with the middle transition at a urea concentration of 3.5 M (Figure 1B). When the ADGroEL was heated, aggregation began at temperatures of above 50 °C (Figure 2), which corresponded to the onset of the thermal denaturation of the protein.

### 2.2. Design and Production of ADGroEL–SacSm Fusion Protein

**Figure 3:**
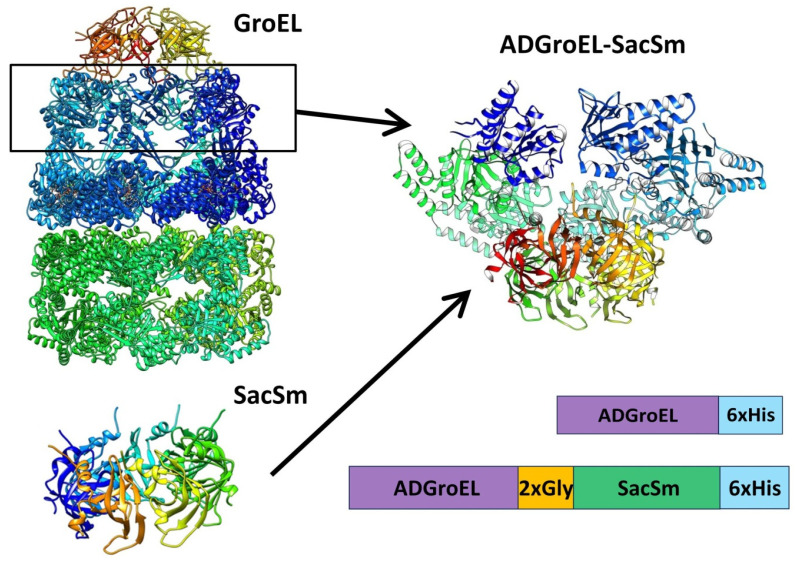
Schematic representation of the ADGroEL–SacSm fusion protein construction based on the GroEL (PDB: 1AON) and SacSm (PDB: 5MKL) structures. Block diagrams represent the resulting protein sequences.

**Figure 4:**
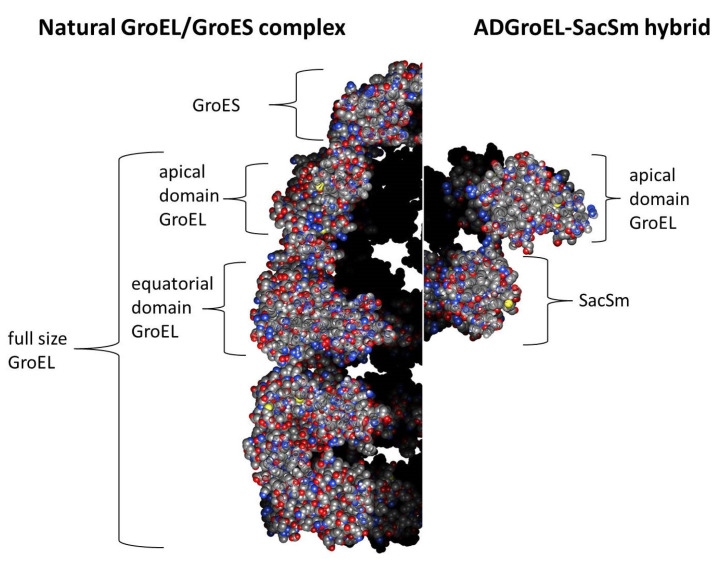
Comparison of the structure, size, and volume of the internal cavity of the natural GroEL/GroES complex (PDB: 1AON) and the hybrid protein ADGroEL–SacSm (calculated structure). Slices of the molecular structures are presented.

The next step was the design of the ADGroEL–SacSm fusion protein (Figure 3) and its production. When designing the fusion protein, we chose a linker made of Gly. Such a linker provides sufficient distance between domains for their stacking into independent rings and, at the same time, is flexible enough for the free mutual motion of domains. A comparison of the structure, size, and volume of the internal cavity of the natural GroEL/GroES complex and the hybrid protein ADGroEL–SacSm is shown in Figure 4. In addition, a His-tag was added to the protein sequence for easy isolation. The genetic constructs were obtained using standard genetic engineering methods. The resulting genetic constructs were expressed in *E. coli* strain BL21 (DE3). Selection of the extraction technique for the most effective purification was carried out. The cells were homogenized in the presence of 6M guanidine. At the same time, our hybrid protein was completely unfolded. The debris was precipitated with centrifugation. Next, the supernatant was applied to a Ni-chelate column. The next stage was washing with a solution of urea. The denaturing effect decreased and the SacSm part of the hybrid protein was more stably folded. The next step was removing the hybrid protein from the column with imidazole. At this point, the released monomers were oligomerized. However, the ADGroEL remained unfolded because the stability of its structure was much lower than that of the SacSm. A further decrease in the urea concentration led to the final folding of the entire hybrid protein. Isolation can also be completely carried out under non-denaturing conditions, but the resulting protein preparation will be more contaminated with impurities.

### 2.3. ADGroEL–SacSm Stability and Folding

Verification of the folding and stability of the ADGroEL–SacSm structure was carried out with spectral methods similar to such verification for individual domains (Figure 1 and Figure 2). The resulting protein had a size corresponding to the folded heptamer. It also had a pronounced CD spectrum (Figure 1C), which indicated a developed secondary structure. Equilibrium unfolding of the protein with urea showed a cooperative transition, matched with that in a separate ADGroEL, indicating that it retained its structure. Taken together, these data confirm the correct folding of ADGroEL, fixed on the basis of SacSm.

### 2.4. Functional Activity of AD Fusion Protein GroEL–SacSm

The final step was to test the function of the ADGroEL–SacSm hybrid protein. The main functions of the chaperone are the binding of non-native proteins and the prevention of their aggregation. We experimentally evaluated the binding of the hybrid protein to some non-native proteins and its ability to prevent the aggregation of other proteins when heated. 

**Figure 5:**
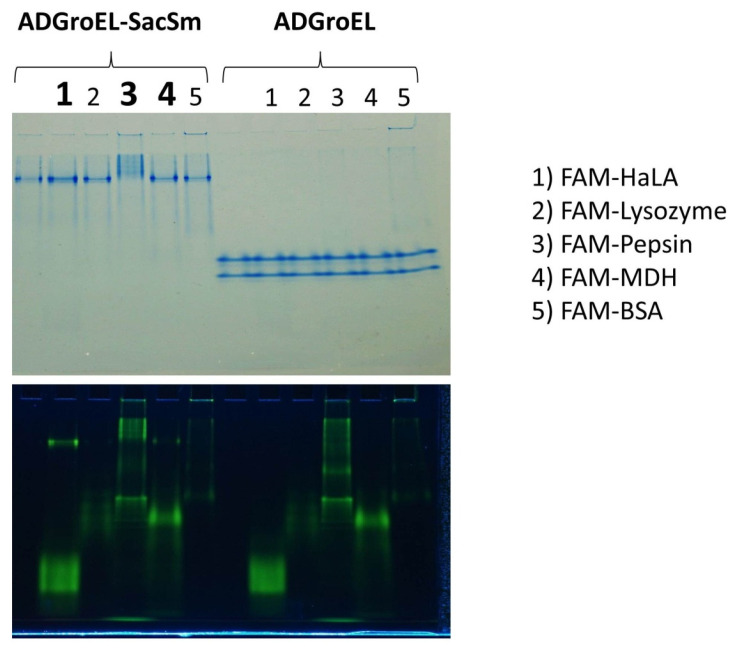
Electrophoretic analysis of the mobility of FAM-labeled non-native proteins in the presences of ADGroEL–SacSm and ADGroEL. Images of the same gel are shown in visible light with Coomassie blue staining (upper panel) and with FAM fluorescence detection (lower panel). Tracks with changes in the mobility of the non-native proteins are signified by bold numbers.

The ability to bind non-native proteins was assessed based on changes in the electrophoretic mobility of fluorescently labeled non-native proteins in non-denaturing electrophoresis. This technique has been actively used by us for the study of chaperone activity and is described in article [10]. According to the results of this experiment, some non-native proteins that bind to full-length GroEL also bound to our fusion protein (Figure 5). At the same time, none of the studied proteins bound to any individual ADGroEL. This suggests that ADGroEL oligomerization is necessary for the strong binding of these proteins. 

**Figure 6:**
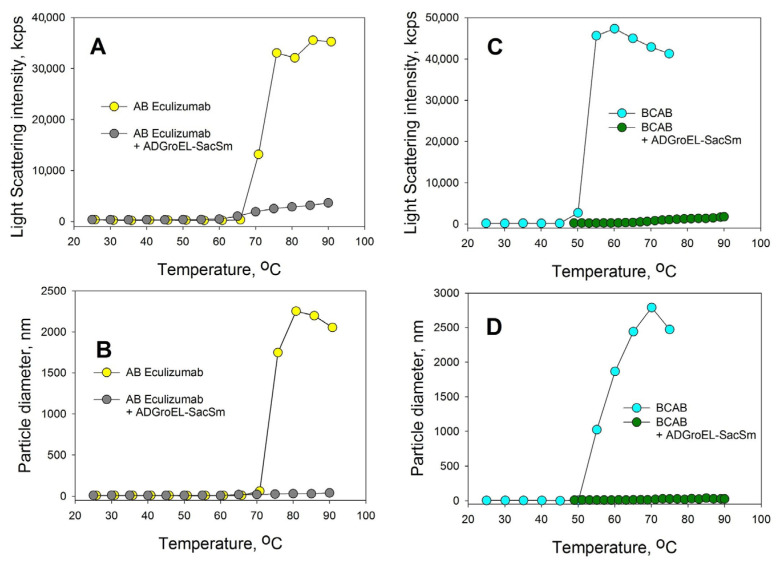
Effects of the ADGroEL–SacSm addition on the aggregations of the eculizumab antibody (**A**,**B**) and carboxyanhydrase B (**C**,**D**). The test protein concentration was 0.2 mg/mL. The molar ratio of the test protein ADGroEL–SacSm was 1:1. The heating was carried out at a rate of 1 °C per minute. The intensity of light scattering (**A**,**C**) and the average particle size in the solution (**B**,**D**) were determined.

The aggregation of proteins upon heating was determined with the DLS method. We chose an Eculizumab antibody and bovine carboxyanhydrase B (BCAB) as model proteins for this study. These proteins were heated separately in the presence of the monomeric ADGroEL and in the presence of the ADGroEL–SacSm fusion protein. The model proteins without additives showed high aggregating tendencies with temperature increases (Figure 6). ADGroEL itself has a tendency to aggregate at temperatures above 50 °C (Figure 2) and therefore could not affect the aggregation of these proteins in any way. Addition of the hybrid protein significantly reduced the light-scattering intensity and the particle size of both test proteins formed during heating. Thus, we can make an unambiguous conclusion that the oligomeric structure coped better with the tasks of binding non-native proteins and preventing their aggregation.

Upon completing the analysis of our data, we could answer the questions asked at the beginning of this article:

(1) How suitable is SacSm as a scaffold for engineering?

It is undoubtedly suitable and quite promising for the creation of new oligomeric proteins.

(2) What features of ADGroEL are retained when it is kept in oligomeric form?

While the oligomeric form of ADGroEL is maintained, its aggregation ability will be significantly reduced, and in addition, the ability to prevent the aggregation of other proteins upon heating will appear. The ability to bind some non-native proteins much more strongly than the monomeric form of the apical domain will also appear.

(3) Will the mutual influence of domains be critical for folding?

The mutual influence of domains on folding in a given protein is not critical. With the correct formulation of the process, complete renaturation of the hybrid protein is possible after unfolding with a strong denaturant. This, however, does not exclude similar problems when other target domains are attached.

## 3. Materials and Methods

### 3.1. Modeling of the Fusion Protein ADGroEL-SacSm 

The program Coot was used for fusion protein model building based on the PDB data of individual proteins. ADGroEL-SacSm fusion protein construction was modeled based on the GroEL (PDB: 1AON) and SacSm (PDB: 5MKL) structures.

### 3.2. Preparation of Genetic Constructs Carrying the Gene That Encodes the ADGroEL Escherichia coli Protein

To obtain the ADGroEL protein, expression vectors based on the vector pET-22b were constructed (pET-22b_ADGroEL). The oligonucleotide primers were synthesized in accordance with the nucleotide sequence of the gene that encodes the ADGroEL *Escherichia coli* (corresponding to amino acid sequence 187D-406V). Forward primer F1 and reverse primer R1 (Eurogen, Moscow, Russia) (Table 1) contain restriction sites for restriction endonucleases NcoI and HindIII (underlined), which are necessary for integrating the target gene into the expression vector pET-22b. Reverse primer R1 contains 6xHis coding sequences (shaded). The genomic DNA of *E. coli* was used as a template. 

### 3.3. Preparation of Genetic Construct Carrying the Gene That Encodes the Fusion Protein ADGroEL–SacSm 

To obtain the fusion protein ADGroEL–SacSm, expression vectors based on the vector pET-22b were constructed (pET-22b_ ADGroEL–SacSm). The gene that encodes the hybrid protein ADGroEL–SacSm was obtained using the method of overlapping regions with four primers and three polymerase chain reactions (PCRs). The oligonucleotide primers were synthesized in accordance with the nucleotide sequences of the genes that encode the SmAP protein of *S. acidocoldarius* (SacSm) and ADGroEL *E. coli*. Forward primer F1 and reverse primer R2 (Eurogen, Russia) (Table 1) were used to amplify the DNA fragment that contains the gene for ADGroEL *E. coli*. The genomic DNA of *E. coli* was used as a template. Forward primer F2 and reverse primer R3 were used in the second PCR for the amplification of the DNA fragment that contains the gene for the SacSm protein. The expression vector pProExHtb contains this gene [8]. Both amplified fragments contained overlapping regions (the sequences of forward F2 and reverse R2 are italicized in Table 1). These overlapping fragments were mixed, denatured, and annealed to obtain a heteroduplex, which was then used as a template in the third PCR. This heteroduplex was amplified using two primers: forward F1 (complementary to the gene for ADGroEL) and reverse R3 (complementary to the gene for the SacSm protein). Forward primer F1 and reverse primer R3 contain restriction sites for the restriction endonucleases NcoI and HindIII (underline), which were necessary for integrating the target gene into the expression vector pET-22b. Reverse primer R3 contains 6xHis coding sequences (shaded). 

### 3.4. Purification of the ADGroEL Protein

The pET-22b vector carrying the ADGroEL protein gene was used for transformation in *E. coli* BL21(DE3) and protein production.

The transformants were grown in an LB medium in the presence of ampicilline (100 µg/mL) at 37 °C with an agitation of 180 rpm. Protein expression was induced at OD_600nm_ = 0.6–0.8 o.u. with the addition of IPTG at a final concentration of 0.5 mM. The bacteria were harvested with centrifugation 3 h after induction.

Cell pellets were suspended in a solution containing 500 mM of NaCl, 50 mM of Tris-HCl (pH 8.0), 10 mM of imidazole, 5 mM of β-mercaptoethanol, and 6 M of Guanidine-HCl. The cells were disrupted with sonication at 4 °C. Cell debris was removed with centrifugation at 15,000× *g* for 30 min at 4 °C. Supernatant was loaded onto a Ni-NTA agarose (GE Healthcare, Uppsala, Sweden) column equilibrated with a solution containing 500 mM of NaCl, 50 mM of Tris-HCl (pH 8.0), 10 mM of imidazole, and 8 M urea. GroELAD_SacSmAP was eluted using a step gradient of imidazole (40 mM and 150 mM) in a solution containing 500 mM of NaCl, 50 mM of Tris-HCl (pH 8.0), and 6 M urea. Fractions containing the protein were collected and purified using Q-Sepharose equilibrated with a solution containing 500 mM of NaCl and 50 mM of Tris-HCl (pH 8.0). Fractions containing the protein were concentrated and dialyzed against a solution containing 150 mM of NaCl and 50 mM of Tris-HCl (pH 8.0). The final purification step was size exclusion chromatography on a Superdex 75 resin equilibrated with a solution containing 150 mM of NaCl and 50 mM of Tris-HCl (pH 8.0). Fractions containing the protein were concentrated and dialyzed against a solution containing 150 mM of NaCl and 50 mM of Tris-HCl (pH 8.0).

### 3.5. Purification of the Fusion Protein ADGroEL_SacSm

The pET-22b vector carrying the ADGroEL_SacSm fusion protein gene was used for transformation in *E. coli* BL21(DE3)/pRARE to express the protein. *E. coli* strain BL21(DE3) cells were preliminarily co-transformed with the pRARE, which carried rare-codon tRNA genes (AUA, AGG, AGA, CUA, CCC, and GGA) to enhance the expression of fusion proteins, including an archaeal SacSm part that contained codons rarely used in *E. coli*.

The transformants were grown in an LB medium in the presence of ampicilline (100 µg/mL) and chloramphenicol (10 μg/mL), at 37 °C with an agitation of 180 rpm. Protein expression was induced at OD_600nm_ = 0.6–0.8 o.u. with the addition of IPTG at a final concentration of 0.5 mM. The bacteria were harvested with centrifugation 3 h after induction.

The procedure for obtaining and isolating of ADGroEL_SacSm was fully similar to that described above for ADGroEL.

### 3.6. Protein Purity Analysis

All stages of protein isolation and purification were controlled with denaturing gel electrophoresis in 12% Tris-Tricine PAGE gel with 1% SDS and tris(2-carboxyethyl)phosphin hydrochloride (TCEP), a disulfide-bound reducing agent. PAGE electrophoresis under non-denaturing conditions in 9% PAGE gel was used to evaluate the homogeneity of the resulting protein under native conditions and to verify whether there were any aggregates in the solution.

### 3.7. Dynamic Light Scattering

The hydrodynamic radii of the particles were estimated with the DLS method using a Zetasizer Nano ZSP instrument (Malvern Instrument Ltd., Malvern, UK). The measurements were carried out at a temperature of 25 °C, and the detection angle was 173°. Quartz cuvettes, each with a volume of 100 μL and an optical path length of 0.3 cm, were used to study the proteins. When the temperature dependence was studied, the heating rate was 1 °C per minute.

### 3.8. CD Spectroscopy

Far ultraviolet (UV) CD spectra were recorded on a Chiroscan spectropolarimeter (Applied Photophysics, Leatherhead, UK) in a cell with an optical path length of 0.1 mm in the wavelength range of 190–250 nm. Molar ellipticity [Θ] was calculated while the concentration of the protein, its amino acid composition, and the dimensions of the cuvette were taken into account.

### 3.9. Non-Denaturation PAGE

Non-denaturation PAGE was performed in linear 10–20% acrylamide gradient gels. A Laemmli buffer system was used for gel electrophoresis. The pH of the stacking gel was increased to 8. 

Protein samples of ADGroEL–SacSm and ADGroEL were prepared by dilution with a buffer containing 50 mM of Tris-HCl at pH 8.0, 10 mM of MgCl_2_, and a 0.2 mg/mL concentration of protein. Urea-denatured FAM-labeled substrate proteins were added to the resulting chaperone solutions. After a few minutes, 10% glycerol with BPB was added and the samples were applied to the gel. The same amount of 8 M urea was added to the samples without substrate proteins.

## 4. Conclusions

The data obtained have opened up large fields of research both in protein engineering using the SacSm protein (and its analogues) as a building block or scaffold and in studying the functioning of chaperones.

The discovered properties of the hybrid protein created by us show the promise of the chosen direction of work. In terms of practical application, this protein has advantages over both monomeric ADGroEL and the full-sized GroEL oligomer. The hybrid is significantly more temperature-resistant and has a higher affinity for non-native proteins than monomeric ADGroEL. At the same time, it is much smaller than full-sized GroEL, which should have a positive effect on its production in cells, and it is much easier to purify.

With this article, we open a series of works on the study of hybrid proteins built on the basis of circular oligomers. Even for the presented protein, a number of questions have arisen that are beyond the scope of this article. How strongly (in comparison to original GroEL) does it bind non-native proteins? Is it capable of promoting proper protein folding (or just binding)? What state are the proteins associated with our hybrid in at temperatures above the melting point? We will devote our further research to answering these and other questions and will undoubtedly present it to readers in our next articles.

## Figures and Tables

**Table 1:** Sequences of oligonucleotides for gene-encoding proteins ADGroEL and ADGroEL–SacSm. Restriction sites for specific restriction endonucleases are underlined, 6xHis coding sequences are shaded, and overlapping regions of forward F2 and reverse R2 are italicized.

Gene Fragment	Primer (Sequence 5′→3′)
ADGroEL	F1 GCGGATCCATGGACGTGGTGAAGGTATGCAG R1 CACCCCAAGCTTTTAGTGGTGGTGGTGGTGGTGGATAACTGCAACGCCGCCTG
ADGroEL–SacSm	F1 GCGGATCCATGGACGTGGTGAAGGTATGCAG R2 *CTATCTTGGCTTGCACTCCTCCTACCGCAGCAC*GGGTCGC
	F2 *GTGCTGCGGTAGGAGGATCAGCCAAGTAG*AAAATC R3 CCCAAGCTTTTAGTGGTGGTGGTGGTGGTGTTTTTCCTTA CCCATTATTGATTC

## Data Availability

Not applicable.
